# Happle–Tinschert, Curry–Jones and segmental basal cell naevus syndromes, overlapping disorders caused by somatic mutations in hedgehog signalling genes: the mosaic hedgehog spectrum

**DOI:** 10.1111/bjd.18150

**Published:** 2019-05-23

**Authors:** M.‐L. Lovgren, Y. Zhou, G. Hrčková, T. Dallos, I. Colmenero, S.R.F. Twigg, C. Moss

**Affiliations:** ^1^ Department of Dermatology Birmingham Children's Hospital Birmingham Women's and Children's NHS Foundation Trust Birmingham U.K; ^2^ Clinical Genetics Group MRC Weatherall Institute of Molecular Medicine University of Oxford John Radcliffe Hospital Oxford U.K; ^3^ Department of Paediatrics Faculty of Medicine Comenius University in Bratislava and National Institute of Children's Diseases Bratislava Slovak Republic; ^4^ Department of Pathology Birmingham Children's Hospital Birmingham Women's and Children's NHS Foundation Trust Birmingham U.K

## Abstract

**Summary:**

Happle–Tinschert syndrome (HTS) and Curry–Jones syndrome (CJS; OMIM 601707) are rare, sporadic, multisystem disorders characterized by hypo‐ and hyperpigmented skin patches following Blaschko's lines, plus acral skeletal and other abnormalities. The blaschkoid pattern implies mosaicism, and indeed CJS was found in 2016 to be caused by a recurrent postzygotic mutation in a gene of the hedgehog signalling pathway, namely *SMO*, c.1234C>T, p.Leu412Phe. More recently the original case of HTS was found to carry the same somatic mutation. Despite this genetic and phenotypic overlap, two significant differences remained between the two syndromes. The histological hallmark of HTS, basaloid follicular hamartomas, is not a feature of CJS. Meanwhile, the severe gastrointestinal manifestations regularly reported in CJS had not been described in HTS. We report a patient whose phenotype was entirely consistent with HTS apart from intractable constipation, and a second patient with classic features of CJS plus early‐onset medulloblastoma, a feature of basal cell naevus syndrome (BCNS). Both had the same recurrent *SMO* mutation. This prompted a literature review that revealed a case with the same somatic mutation, with basaloid follicular hamartomas and other features of both CJS and BCNS. Segmental BCNS can also be caused by a somatic mutation in *PTCH1*. We thus demonstrate for the first time phenotypic and genetic overlap between HTS, CJS and segmental BCNS. All of these conditions are caused by somatic mutations in genes of the hedgehog signalling pathway and we therefore propose the unifying term ‘mosaic hedgehog spectrum’.

**What's already known about this topic?**

- Happle–Tinschert syndrome (HTS) and Curry–Jones syndrome (CJS) are rare mosaic multisystem disorders with linear skin lesions.
- CJS is characterized by severe constipation, which has not previously been reported in HTS.
- HTS is characterized by basaloid follicular hamartomas, which are not a recognized feature of CJS.
- The recurrent mosaic *SMO* mutation found in CJS was recently reported in a patient with HTS.

**What does this study add?**

- We describe a patient with HTS and intractable constipation, and a case of CJS with medulloblastoma.
- Both patients had the same recurrent somatic *SMO* mutation also found in a case reported as segmental basal cell naevus syndrome.
- *SMO* functions in the hedgehog pathway, explaining phenotypic overlap between HTS, CJS and mosaic basal cell naevus syndrome.
- We propose the term ‘mosaic hedgehog spectrum’ for these overlapping conditions.

## Abstract 2

**Linked Comment:** https://doi.org/10.1111/bjd.18416.

Curry–Jones syndrome (CJS; OMIM 601707) is characterized by, among other things, acral skeletal anomalies and hyper‐ and hypopigmented skin lesions following Blaschko's lines. These two features also occur in Happle–Tinschert syndrome (HTS). In 2016, Twigg *et al*. identified a recurrent postzygotic mosaic mutation in the smoothened gene (*SMO*), c.1234C>T, encoding p.Leu412Phe, in eight cases of CJS.^1^ Prompted by the phenotypic overlap, Zenker *et al*. found the same mutation in their original patient with HTS.^2^ There are significant differences between the two syndromes: the HTS skin lesions are typically basaloid follicular hamartomas (BFHs), which are not a known feature of CJS. Meanwhile, severe constipation characterizes CJS but is not reported in HTS. Furthermore, both have features in common with a third syndrome, mosaic basal cell naevus syndrome (BCNS).

We report two new patients with the same recurrent mutation in *SMO*. One has classic features of HTS with, in addition, lifelong intractable constipation. The second has classic features of CJS plus medulloblastoma. These informative patients clarify the links between the two disorders.

## Case report

### Case 1

**Figure 1:**
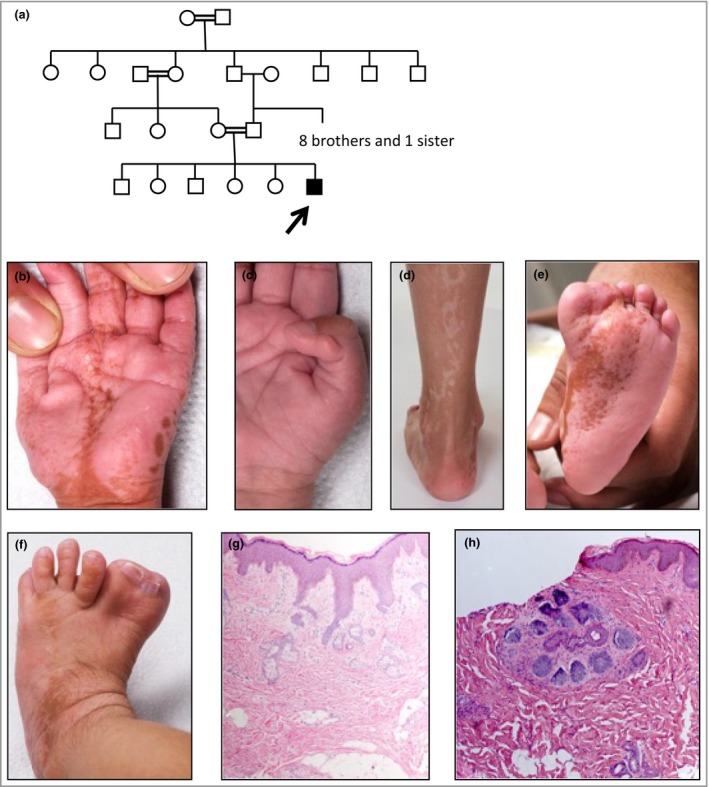
Pedigree, clinical images and skin histology of case 1. (a) Pedigree of case 1. The arrow indicates the proband. (b) Hypoplastic left thumb with linear hyperpigmented pitted lesions on the palm. (c) Rudimentary right thumb. (d) Linear hypopigmented streaks on the posterior left calf. (e) Linear hyperpigmented pitted lesions on the left sole. (f) Duplication and syndactyly of the left great toe. Hyperpigmented lesions with overlying hypertrichosis on the dorsum of the foot. (g) Biopsy from a hyperpigmented lesion on the left sole (e), demonstrating small buds and nests of basaloid cells arising from the epidermis with peripheral palisading, surrounded by a rim of hyalinized stroma. Within the dermis are abnormally formed sebaceous glands not associated with hairs. The features are in keeping with basaloid follicular hamartomas. Haematoxylin and eosin (HE), original magnification × 100. (h) Biopsy from a hypopigmented lesion on the left calf (d), demonstrating a nodule with nests of basaloid proliferation within its own prominent stroma in the dermis, not connected to the epidermis. The features are consistent with a trichoblastoma. HE, original magnification × 100.

A 9‐year‐old boy was born at term to consanguineous parents of Pakistani origin (Fig. 1a).^3^ He had asymmetrical congenital anomalies: rudimentary right thumb duplication, left hypoplastic thumb, great toe duplication and syndactyly, and shortened leg. There were left‐sided whorled, hypopigmented lesions, some atrophic and others palpable, following Blaschko's lines on the trunk and limbs. Linear pitted hyperpigmentation affected the left palm and sole, with naevoid hypertrichosis on the dorsal foot and thigh (Fig. 1b–f). Histology of affected skin was initially reported as basal hyperpigmentation, but on review revealed BFHs (Fig. 1g) and a trichoblastoma (Fig. 1h), which was consistent with HTS.

He experienced lifelong constipation that was resistant to high‐dose laxatives. Rectal biopsies and barium meal excluded Hirschsprung disease and malrotation. Abdominal radiograph and magnetic resonance imaging demonstrated large bowel distension.

DNA microarray analysis revealed a single bacterial artificial chromosome clone duplication at 5p15·2^4^ in affected skin, but not in his or his parents’ blood. Higher‐resolution oligonucleotide microarray analysis did not confirm the duplication. It was considered unlikely to be clinically relevant.

**Figure 2:**
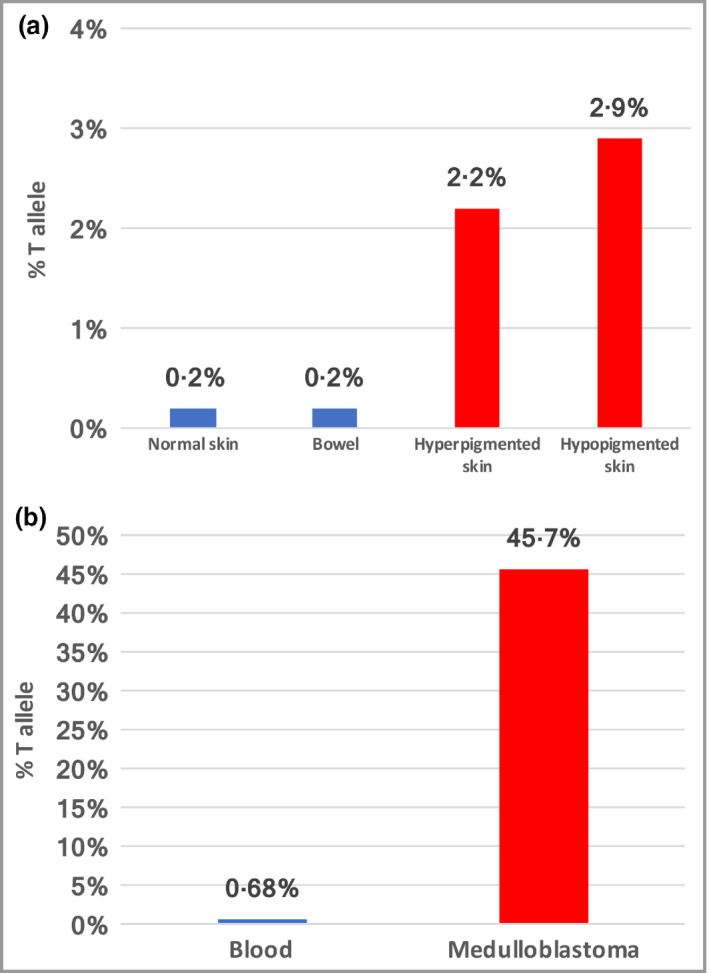
(a) Case 1: proportion of *SMO* c.1234C>T, Leu412Phe mutant T allele in the affected hyper‐ and hypopigmented skin (red bars) and clinically unaffected skin and bowel (blue bars). (b) Case 2: proportion of *SMO* c.1234C>T, Leu412Phe mutant T allele in the medulloblastoma (red bar) and blood (blue bar). Skin tissue was not available for genetic analysis.

Deep sequencing of *SMO* (Appendix S1; see Supporting Information) demonstrated the recurrent gain‐of‐function mutation c.1234C>T at 2·2% and 2·9% in hyper‐ and hypopigmented skin, respectively. The 0·2% level found in normal skin and bowel is indistinguishable from controls (data not shown); < 1% was deemed clinically insignificant (Fig. 2a).

This finding of the CJS mutation prompted review of his gastrointestinal problems. In the absence of malrotation or hamartomas, which are sometimes described in CJS, his constipation was attributed to dysmotility (‘pseudo‐obstruction’), also reported in CJS. Rectal washouts have been commenced and colostomy is being considered.

### Case 2

**Figure 3:**
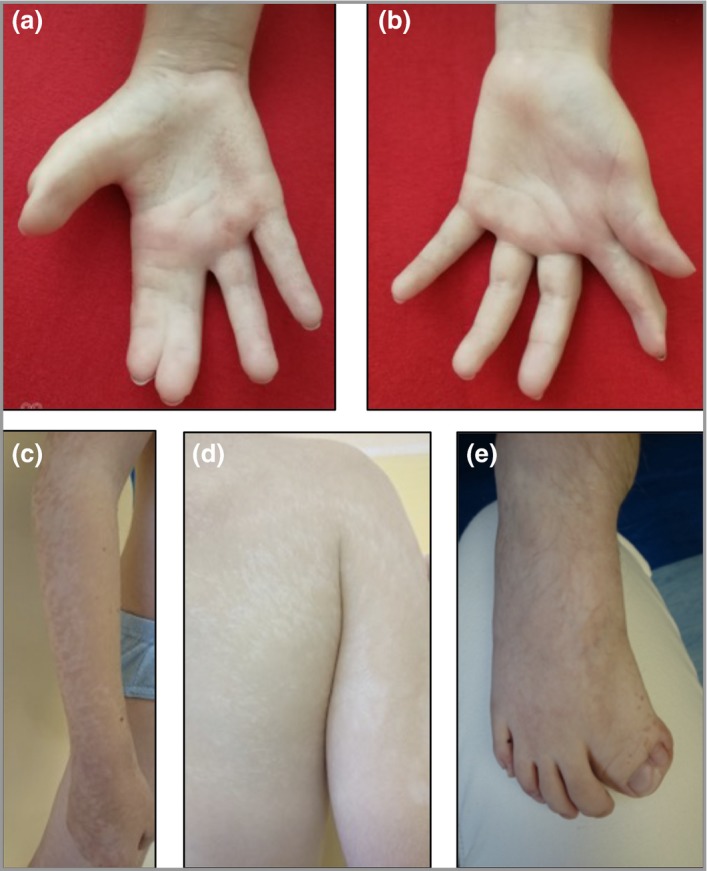
Clinical images of case 2. (a) Right second‐ and third‐finger syndactyly with short digits and hyperpigmented pitted lesions. (b) Reconstructed left first‐ and second‐finger syndactyly with brachyphalangy. (c) Right arm: linear atrophic hypopigmentation. (d) Right back and upper arm: linear atrophic hypopigmentation. (e) Right great toe duplication and syndactyly with overlying hyperpigmented lesions and hypertrichosis on the dorsum of the foot.

A male infant was born to nonconsanguineous parents of Slovak origin, with right‐sided atrophic linear hypopigmentation on the trunk and limbs and hyperpigmented pitted palmar lesions. Skeletal anomalies included right 2–3 and left 1–2 finger syndactyly, short digits and right great toe duplication with a rudimentary phalanx (Fig. 3). He also had right coloboma iridis, multiple pigmented intradermal naevi, lentigines and retained primary dentition, with two rows of teeth.

Aged 2 years, a posterior fossa medulloblastoma, World Health Organization grade IV, was successfully treated. Aged 4 years, he presented acutely with pseudo‐obstruction. Magnetic resonance enterography showed small bowel nodularity and hepatic haemangiomas. Bowel resection demonstrated small bowel leiomyomas.

Aged 12 years, his height and weight remain between the 0·4th and 2nd centiles. The recurrent *SMO* mutation c.1234C>T was identified in the medulloblastoma (45·7% mutant *SMO* reads), and at insignificant levels in the blood (Fig. 2b). The skin was not biopsied. He was diagnosed with CJS.

## Discussion

HTS and CJS are rare sporadic multisystem disorders. Curiously, unlike CJS, HTS has never been recognized with a McKusick number. Since 1995,^5^ 13 cases of CJS have been reported, with the causal mosaic *SMO* c.1234C>T mutation identified in 2016.^1^ HTS was described in 2008, with around 14 reported cases. The genetic basis for HTS was unknown until 2018, when Zenker *et al*. demonstrated the identical mosaic *SMO* mutation in Happle's original patient with HTS.^2^ Our first case is only the second report of *SMO* mutation in HTS, confirming genetic overlap between HTS and CJS.

HTS and CJS also have significant phenotypic overlap, principally cutaneous and skeletal manifestations, but also cerebral, craniofacial, ophthalmological and dental abnormalities (Table 1). The discrepant features are BFHs only reported in HTS, and gastrointestinal manifestations cardinal to CJS.^6^ Case 1 has both differentiating features: BFHs and constipation.

**Table 1:** Clinical features of Happle–Tinschert syndrome (HTS), Curry–Jones syndrome (CJS) and basal cell naevus syndrome (BCNS)

Clinical features	HTS	CJS	BCNS
Mutation	Somatic *SMO* c.1234C>T	Somatic *SMO* c.1234C>T	*SMO* and *PTCH1*
Cutaneous	Basaloid follicular hamartomas		Basaloid follicular hamartomas
	**Linear hypo‐ or hyperpigmented lesions**	**Linear hypo‐ or hyperpigmented lesions**	**Linear hypo‐ or hyperpigmented lesions**
	**Palmoplantar pitting**	**Palmoplantar pitting**	**Palmoplantar pitting**
	**Atrophoderma**	**Atrophoderma**	**Atrophoderma**
	**Hypertrichosis**	**Hypertrichosis**	**Hypertrichosis**
	Basal cell carcinoma	Trichoblastoma	Basal cell carcinomas
	Hypotrichosis	Naevus sebaceous	
Skeletal	**Polydactyly or syndactyly**	**Polydactyly or syndactyly**	**Polydactyly or syndactyly**
	Rib anomalies (rudimentary ribs)		Rib anomalies: bifid, splayed, extra rib
	Limb‐length anomalies		Vertebral anomalies (bifid)
Craniofacial	**Dysmorphic facies**	**Dysmorphic facies**	**Dysmorphic facies**
	**Macrocephaly**	**Macrocephaly**	**Macrocephaly**
	Jaw tumour (ameloblastoma)	Microcephaly	Jaw tumour (keratocyst)
	Dental anomalies	Dental anomalies	Calcification of falx cerebri
		Craniosynostosis	Cleft lip/palate
Gastrointestinal	Anal anomaly (imperforate)	Anal anomaly (stenosis)	
	Colonic adenocarcinoma	Severe constipation	Severe constipation
		Myofibromas and smooth muscle hamartomas	
		Malrotation	
Cerebral	**Medulloblastoma**	**Medulloblastoma**	**Medulloblastoma**
	Cerebral malformations	Cerebral malformations	
	Developmental delay	Developmental delay	
	Optic glioma or meningioma		
Ophthalmic	**Cataract**	**Cataract**	**Cataract**
	**Microphthalmia**	**Microphthalmia**	**Microphthalmia**
	**Coloboma**	**Coloboma**	**Coloboma**
		Glaucoma	Glaucoma, developmental defects
Gonadal		Cryptorchidism	Ovarian fibromas
Other			Cardiac fibromas

Eleven features found in all three syndromes are bold. Of the 39 features listed, 15 are shared between HTS and CJS, 12 between CJS and BCNS, and 15 between HTS and BCNS.

BFHs are benign hair follicle cell tumours presenting as atrophic or papular hypo‐ or hyperpigmented lesions with comedo‐like plugs. Brown, punctuate lesions on plantar surfaces appear to be characteristic of HTS, and histologically show BFH. Patients with CJS show clinically similar linear hypo‐ or hyperpigmented lesions, including palmoplantar pitting. One case of CJS had biopsy‐confirmed trichoblastomas,^7^ similar to our case, and another was reported with a naevus sebaceous.^5^ BFHs have not been reported in CJS, but our first case demonstrates that they can be missed.

Both of our cases demonstrated tissue mosaicism. Mutation levels in affected skin were low, possibly because DNA was extracted from dermal fibroblasts, while the abnormality is epidermal. The absence of mutation in bowel from case 1 may simply reflect a patchy distribution of affected cells, and we suspect the bowel sample was from an unaffected area. As the levels of *SMO* mutation in unaffected skin and blood were also below 1% these were considered negative, as described previously.^1^ In case 2, the *SMO* mutation was evident in medulloblastoma tissue, but other tissues were not tested. The clinical phenotype strongly suggests widespread *SMO* mosaicism, although this remains unproven.

Khamaysi *et al*. reported the same mosaic *SMO* mutation in a patient they considered to have segmental BCNS, as he fulfilled the diagnostic criteria, but whose features were interpreted by Happle and Tinschert as HTS, suggesting this is a variant of CJS.^8^, ^9^ This patient had linear skin‐coloured or pigmented lesions, comedones, pits, shortened digits and multiple basal cell carcinomas (BCCs). Interestingly he developed bowel perforation due to constipation. A palmar pit biopsy was suggestive of BFH. Khamaysi *et al*. argued that multiple BCCs were consistent with BCNS but not HTS.^10^ Several older reports described BCCs in patients with features suggestive of HTS,^11^, ^12^, ^13^ while basaloid hamartomas have been described in classic and segmental BCNS with *PTCH1* mutations. Torrelo *et al*. reported a girl with paternally inherited BCNS and mosaicism for a second *PTCH1* mutation, with asymmetrical palmar pits, hyper‐ and hypotrichosis, skeletal abnormalities and hyper‐ and hypopigmented lesions, confirmed to be BCCs and a basaloid hamartoma.^14^

Both our cases demonstrate features associated with BCNS. In case 1, palmar and plantar pits are a major criterion for BCNS, and polydactyly is a minor feature; radiographs did not show other skeletal BCNS manifestations. Case 2 had sufficient criteria to diagnose BCNS, having pitted palmar lesions (major criteria), with polydactyly and medulloblastoma (minor criteria). No calcification of the falx cerebri or vertebral abnormalities were detected on X‐ray imaging. Thus, there is phenotypic overlap between the three conditions: HTS, CJS and BCNS.

The somatic *SMO* c.1234C>T mutation has been identified in isolated cases of BCC, ameloblastoma, meningioma and medulloblastoma. These tumours also occur in HTS and CJS.^7^, ^15^ The *SMO* mutation leads to constitutive activation of the hedgehog–patched–GLI pathway,^1^ with resultant cell proliferation and tumorigenesis. This explains the similarity of HTS and CJS to BCNS, which is usually caused by upstream heterozygous *PTCH1* inactivating mutations that result in *SMO* activation. These three conditions – HTS, CJS and segmental BCNS – could be considered a spectrum of mosaic hedgehog disorders caused by postzygotic mutations in hedgehog signalling genes. The phenotypic heterogeneity, despite a shared molecular basis, is likely due to timing of the mutation, tissue distribution and the consequent mutant load. Identification of the *SMO* mutation offers new therapeutic possibilities including vismodegib, an *SMO* inhibitor. Recognition of genetic overlaps between disparate conditions facilitates a fresh look at symptoms and therapeutic options.

## Supporting information


**Appendix S1** *SMO* sequencing method.
