# Supplementary material for: Happle–Tinschert, Curry–Jones and segmental basal cell naevus syndromes, overlapping disorders caused by somatic mutations in hedgehog signalling genes: the mosaic hedgehog spectrum
Source: Br J Dermatol. 2019 May 23;182(1):212–7. doi: 10.1111/bjd.18150 (PMC6972552; doi:10.1111/bjd.18150)
Supplement: Supplementary file 1 — Appendix S1 SMO sequencing method. [file BJD-182-212-s001.docx]

Supporting Information

File 1.

***SMO* sequencing method**

*SMO* exon 6 was amplified using template-specific primers that were tagged with barcoded (6 nucleotide indexes) Illumina adapter sequences to generate products of approximately 200 bp. Reactions used 0.02 U/µl of high fidelity Taq polymerase Q5 (NEB) in a volume of 25 µl containing, 40 ng DNA, 0.5 µM primers, 25 mM Tap-HCl (pH 9.3), 50 mM KCl, 2 mM MgCl_2_, 1 mM ß-mercaptoethanol and 200 µM each dNTP. Cycling consisted of 30 s of denaturation at 98°C, followed by 15 cycles of 98°C for 10 s, 64°C for 20 s with a 0.5°C decrease each cycle and 72°C for 30s, and then a further 15 cycles of 98°C for 10 s, 57°C for 20 s and 72°C for 30s, with a final extension at 72°C for 2 min. Barcoded products were pooled, then purified using Axyprep beads (Axygen) and sequenced to a minimum depth of 14,000 using an Illumina MiSeq 300v2 kit according to the manufacturer’s instructions. The forward primer was used in conjunction with one of the barcoded reverse primers per sample:

Forward oligo (*SMO* specific sequences underlined):

PE1 Smo_ex6F:

AATGATACGGCGACCACCGAGATCTACACTCTTTCCCTACACGACGCTCTTCCGATCTGTGGGCTACAAGAACTACCGATACCGTG

Reverse barcoded oligos (*SMO* specific sequences underlined; barcodes in lowercase):

PE2_BC11_Smo_ex6R:

CAAGCAGAAGACGGCATACGAGATgtagccGTGACTGGAGTTCAGACGTGTGCTCTTCCGATCTCTGGTCCTGGCCTGGTCTTCACTCAC

PE2_BC12_Smo_ex6R:

CAAGCAGAAGACGGCATACGAGATtacaagGTGACTGGAGTTCAGACGTGTGCTCTTCCGATCTCTGGTCCTGGCCTGGTCTTCACTCAC
